# Supplementary material for: Impact of a mobile decision support tool on antimicrobial stewardship indicators in St. John’s, Canada
Source: PLoS One. 2021 Jun 16;16(6):e0252407. doi: 10.1371/journal.pone.0252407 (PMC8208547; doi:10.1371/journal.pone.0252407)
Supplement: Attachment — Submitted filename: plos one review-AMS_spectrum..pdf [file pone.0252407.s003.pdf]

Abstract
1. Recommend to add the background of study with the problem statement in the abstract section.
2. Suggest further description on the methods in the abstract section. The statement of "We performed two one-day inpatient point prevalence surveys using the National Antimicrobial Prescribing Survey tool, before (June 25, 2018) and six months after (June 25, 2019) app dissemination to prescribers" is unclear. When is exactly the implementation date of the SpectrumTM and how long is the study period?
3. Recommend the authors to provide a careful study conclusion especially by inferring the direct impact/causal relationship of SpectrumTM in improving AMS indicators (in the abstract section).

Introduction
1. Recommend further explanation and elaboration on: AMU, AMR, AMS, SpectrumTM and the outcome measures ie. appropriateness of antimicrobial prescribing, AMU and CDI. Although the authors had explained on the SpectrumTM, it would be better if they could also explain on the previous evaluation or validation conducted on the apps.
2. Page 3, line 54-56 - Recommend the authors to revise this statement: "A mobile decision support application ("app") improved guideline adherence among patients admitted with respiratory infections in New Zealand by eight percent, compared to no improvement in a control hospital (p=0.04)". Based on the reference cited, the mobile apps improved guideline adherence by the prescriber, but not the guideline adherence among patients...
4. Page 4, line 72 - The objectives statement is unclear especially for "..... and inpatient appropriateness....." - suggest to revise.

Methods
1. Page 4, line 78 - Recommend to describe clearly on the study setting. What do you mean by "both tertiary-care academic hospitals....."?
2. Kindly clarify the duration of study: 1 year (25 June 2018 to 25 June 2019 OR January 2019 to March 2020)?
3. Recommend the authors to revise the methods section to increase clarity and allow reproducibility.
4. Suggest the authors to revise the subtopic for the methods. eg. Appropriateness - In what aspect/scope?
5. Recommend to further describe the statistical analysis and need to correspond to the results section.

Results
1. Recommend further description on the costing in methods and data/analysis in the manuscript.
2. Suggested to revise the statements on the appropriateness (page 14, line 156-158).
3. Recommend further elaboration/description for statement ; page 14, line 162-163.

4. Suggest the author to improve the presentation of results. It would be better not in a form of graph or bar chart only, but in a form of table might further increase readers understanding. The current bar charts are not self-explanatory. There are quite number of missing details/information.
5. Suggest the author to improve Table 1 - How did you get the p value ie. using what statistical test.
6. Overall, recommend the author to revise the results section of the manuscript to increase readers' understanding.

Discussion
1. Suggested that the Discussion section contain specific recommendations for future research to improve AMS outcomes.
2. Recommend the authors to elaborate on the inpatient bed-days vs. inhabitant-days and purchasing-level data. Please explain the concerns in detailed. How do they affect the findings?
3. Page 17, line 231 - 235 - suggest the authors to discuss more on the statements.
4. Discussions were more on the appropriateness of antimicrobial prescribing.
5. Recommend to discuss also on the utilization and CDI.
6. Suggest the authors to add conclusion section.
