# Supplementary material for: Impact of a mobile decision support tool on antimicrobial stewardship indicators in St. John’s, Canada
Source: PLoS One. 2021 Jun 16;16(6):e0252407. doi: 10.1371/journal.pone.0252407 (PMC8208547; doi:10.1371/journal.pone.0252407)
Supplement: Attachment — Submitted filename: PLoS Response to Reviewers April 29.docx [file pone.0252407.s005.docx]

Public Library of Science

Response to Reviewers

Impact of a Mobile Decision Support Tool on Antimicrobial Stewardship Indicators in St. John’s, Canada

April 29, 2021

Dear Editor,

Thanks for your thoughtful peer review on our submitted article. We respond with a revised version, including response to each suggestion below.

Response to Reviewer comments:

Reviewer #2: Most of the concerns raised by the reviewers were well and properly addressed by the authors. Thank you. However, there are still minor amendments required as suggested below:

1. There are information on the cost saving from the implementation of the SpectrumTM app, although it was not stated in the objective of the study. There is no details on how the costing were done or calculated. Since the study objectives did not include cost, the authors may just omit the component in the manuscript.

*Thanks, we have added cost analysis to the objectives of the study, and further information on cost analysis methods.*

2. Please address the reviewer suggestion to include in the discussion section: "Would consider commenting on results of a randomized study that evaluated the impact of a clinical decision support tool on clinical outcomes (i.e., length of stay, 30- day mortality, 30-day re-admission) and C. difficile infection, acquisition of multidrug resistant organisms, and antibiotic cost (reference: Ridgway JP, Robicsek A, Shah N, et al. Clinical Infectious Diseases 2020).

*Thanks for this suggestion, we had included this reference in the introduction section and have now added discussion comparing our results to this reference. This study used a different decision support intervention and did not demonstrate reduction in CDI.*
